# Supplementary material for: Mutations in Components of the TREX‐2 Complex Result in Misexpression of the Kelch‐Domain F‐Box Protein KFB39 Promoter in Arabidopsis thaliana
Source: Plant Direct. 2026 Jan 30;10(2):e70139. doi: 10.1002/pld3.70139 (PMC12856525; doi:10.1002/pld3.70139)
Supplement: Supplementary file 1 — Data S1: Peer Review. [file PLD3-10-e70139-s003.pdf]

Mutations in components of the TREX-2 complex result in misexpression of the Kelch-domain F-Box protein KFB39 promoter in Arabidopsis thaliana.

Johnathan Napier; Michaela C Matthes; Smita Kurup; John A Pickett

Decision Letter Round 1:

January 8, 2026

Prof. Johnathan Napier

Rothamsted Research

Rothamsted Research, Crop Performance and Improvement

Harpenden, N/A AL5 2JQ

United Kingdom

RE: Mutations in components of the TREX-2 complex result in misexpression of the Kelch-domain F-Box protein KFB39 promoter in Arabidopsis thaliana

Dear Jonathan:

Thank you for submitting to Plant Direct. All required reviews have been returned and we have now finished our evaluation of your manuscript. In light of the reviewers' and editor's comments, further revisions are needed before the paper can be accepted for publication in Plant Direct.

Please view the editors' and reviewers' comments below and use their suggestions as a guide while you work on your revision.

When uploading the revised version of this article, please be sure to include the following:

-A word document that contains your response to the reviewers. You should respond to each reviewer comment and note the changes made to the manuscript. If you do not agree with a reviewer's comment and choose not to make a suggested revision, please explain why. Please try to provide as complete an answer as possible to each reviewer's criticisms.

-A tracked changes document with each change highlighted

- A clean version of the latest version of the manuscript

To upload your revision, please click the link below.

https://plantdirect.msubmit.net/cgi-bin/main.plex?el=A7Lr3qB1A2qp7I7A9ftdtAPJW90cYPgntm0vFPLL1wZ

In order to provide as timely a service as possible, we ask that your revision is resubmitted within three months after receipt of this request. If an extension is needed, please send a request, along with a brief explanation, to the editorial office at plantdirect@wiley.com .

Please note that, in addition to publishing reviewer comments, the author's responses to review comments will also be published alongside the final version of the paper. If you would not like the author's responses to be published, please contact the editorial office at plantdirect@wiley.com .

Thank you very much for giving us an opportunity to review your work. I look forward to receiving the next version.

Sincerely,

Jonathan Ingram

Editor, Plant Direct

--- Editor comments:

Based on my own reading and the important comments of the reviewer, this will make a valuable contribution to Plant Direct.

Please pay attention to the points raised by the reviewer, and revised accordingly.

Please also ensure figures are provided without legends, as these should be editable text.

--- Reviewer comments:

Reviewer #1:

Through a forward genetic screen using a cis-jasmone-responsive KFB39 promoter-GUS reporter system, the authors identified two new alleles (atsac3b-4 and atthp1-5) affecting components of the TREX-2 nuclear pore complex, which result in mis-expression of the KFB39 promoter-GUS cassette.

The authors present phenotypic analyses of the atsac3b-4 and atthp1-5 mutants and demonstrate effects on miRNA localization and/or distribution. However, a functional link between deficiencies in these two TREX-2 components and the mis-expression of

the KFB39 promoter-GUS reporter is not clearly established. The finding that specific nuclear pore complex components influence KFB39 expression is interesting; nevertheless, the data presented are largely descriptive and lack in-depth mechanistic analysis. As such, the work may be somewhat premature. The authors proposed several possible mechanisms that could connect SAC3B and THP1 to KFB39 gene regulation, which are worthy of experimental exploration.

Minor issue:

The KFB39 promoter-GUS system described here appears to show little or no expression in normally growing wild-type plants and is induced only by cis-jasmone. This expression pattern differs from previously reported KFB39 expression profiles in the literatures. It would therefore be helpful to describe the structure and sequence features of the KFB39 promoter (-GUS construct) more explicitly in the methods section or as supplementary information.

Response Letter Round 1:

Plant Direct: 2025-01857

Mutations in components of the TREX-2 complex result in misexpression of the Kelchdomain F-Box protein KFB39 promoter in Arabidopsis thaliana

Matthes et al.

Response to Reviewer's comment

Editor comments:

Based on my own reading and the important comments of the reviewer, this will make a valuable contribution to Plant Direct.

□ Thanks! This manuscript represents over 15 years' worth of effort to define the role of TREX-2 in the regulation of KFB39 and we believe that it could be of interest to the research community.

Please pay attention to the points raised by the reviewer, and revised accordingly.

Please also ensure figures are provided without legends, as these should be editable text.

□ Now corrected – the legends have been removed from the Figures and instead are solely presented in the main manuscript text.

Reviewer comments:

Reviewer #1:

Through a forward genetic screen using a cis-jasmone-responsive KFB39 promoter-GUS reporter system, the authors identified two new alleles (atsac3b-4 and atthp1-5) affecting components of the TREX-2 nuclear pore complex, which result in mis-expression of the KFB39 promoter-GUS cassette.

The authors present phenotypic analyses of the atsac3b-4 and atthp1-5 mutants and demonstrate effects on miRNA localization and/or distribution. However, a functional link between deficiencies in these two TREX-2 components and the mis-expression of the KFB39 promoter-GUS reporter is not clearly established. The finding that specific nuclear pore complex components influence KFB39 expression is interesting; nevertheless, the data presented are largely descriptive and lack in-depth mechanistic analysis. As such, the work may be somewhat premature. The authors proposed several possible mechanisms that could connect SAC3B and THP1 to KFB39 gene regulation, which are worthy of experimental exploration.

Minor issue:

The KFB39 promoter-GUS system described here appears to show little or no expression in normally growing wild-type plants and is induced only by cis-jasmone. This expression pattern differs from previously reported KFB39 expression profiles in the literatures.

 Our previous publications (Matthes et al., 2010; Curtis et al., 2013) show using both northern blotting and promoter-GUS fusions that KFB39 is not expressed during most of the Arabidopsis life cycle. One paper that we cite (Zhang et al., 2015 10.1104/pp.114.249136) shows low expression of KFB39 in 5 day old seedlings, although it should be noted that these plants were being subject to stress to induce

the accumulation of phenylpropanoids.

It would therefore be helpful to describe the structure and sequence features of the KFB39

promoter (-GUS construct) more explicitly in the methods section or as supplementary

information.

 We have now added a full description of the KFB39 promoter-GUS construct into the

Materials and Methods – apologies for not including that before. Hopefully, this

information, in conjunction with Supplementary Table S1, is now more clear.

Decision Letter Round 2:

January 12, 2026

Prof. Johnathan Napier

Rothamsted Research

Rothamsted Research, Crop Performance and Improvement

Harpenden, N/A AL5 2JQ

United Kingdom

MSID: 2025-01857R1

MS TITLE: Mutations in components of the TREX-2 complex result in misexpression of the Kelch-domain F-Box protein KFB39 promoter in Arabidopsis thaliana

Dear Johnathan:

I am pleased to inform you that your manuscript "Mutations in components of the TREX-2 complex result in misexpression of the Kelch-domain F-Box protein KFB39 promoter in Arabidopsis thaliana" has been accepted for publication in Plant Direct.

Your article will appear online in the next available issue of Plant Direct. To ensure your article gets published as quickly as possible, please pay attention to the steps detailed below. We have found that most of the delays happen at this stage, especially at the payment stage, so please respond as quickly as possible when prompted.

License Agreement: Once your article has been accepted it will move to Production and undergo admin and file checking - you may receive an email with any queries we have at this stage. When all required items are received by the publisher and queries resolved, the corresponding author will receive an email from Wiley's Author Services system which will ask them to log on at https://authorservices.wiley.com/bauthor and will present them with the appropriate license for completion. Your article cannot be published until both the signed license agreement and payment of the article fee have been received.

Payment of the Open Access Article Publication Fee: All articles published in Plant Direct are fully open access: immediately and freely available to read, download and share. Plant Direct charges a publication fee to cover the publication costs. The corresponding author for this manuscript should have already received a quote from the payments team (cs-openaccess@wiley.com) with the estimated article publication fee; please email cs-openaccess@wiley.com if this has not been received. The corresponding author should log on to the Wiley Author Services site, where the publication fee can be paid by credit card or an invoice. Pro Forma can also be requested. Payment of the publication charge must be received before the article will be published online.

Proofs: You will have the opportunity to look over your paper once more when you receive the author proofs for your article. The proofs will be with you in approximately two weeks. Please note that, in addition to publishing reviewer comments, the author's responses to review comments will also be published alongside the final version of the paper. If you would not like the author's responses to be published, please contact the editorial office at plantdirect@wiley.com .

Promotion of your article: You can help your research get the attention it deserves! Check out Wiley's free Promotion Guide for best-practice recommendations for promoting your work at www.wileyauthors.com/eeo/guide. And learn more about Wiley Editing Services which offers professional video, design, and writing services to create shareable video abstracts, infographics, conference posters, lay summaries, and research news stories for your research at www.wileyauthors.com/eeo/promotion.

Thank you again for your contribution to Plant Direct. If you have any questions, feel free to contact the editorial office at plantdirect@wiley.com .

Best wishes,

Jonathan

Dr Jonathan Ingram

Editor, Plant Direct
